# Soy Flour and Radish Leaf-Enriched Steamed Dumplings (Manti): Technological, Nutritional, and Sensory Characteristics

**DOI:** 10.3390/foods15020243

**Published:** 2026-01-09

**Authors:** Yurii Syromiatnykov, Shakhista Ishniyazova, Dildora Nurvafaeva, Zuxra Saidmuradova, Abdusator Yusupov, Giyos Tursunov, Ulmas Safarov, Shaxnoza Shamsieva, Shuxrat Yusupov

**Affiliations:** 1 Institute of Soil and Plant Sciences, Latvia University of Life Sciences and Technologies, 3001 Jelgava, Latvia; 2 Department of Fruit and Vegetable Growing and Viticulture, Institute of Agrobiotechnologies and Food Safety, Samarkand State University Named after Sharof Rashidov, Samarkand 140104, Uzbekistan; 3 Department of Agricultural Engineering, Faculty of Mechatronics and Engineering, State Biotechnological University, 61002 Kharkiv, Ukraine; 4 Department of Agricultural Engineering, Karshi State Technical University, Karshi 180100, Uzbekistan; yusupovshuxrat76@gmail.com; 5 Department of Production, Storage and Processing of Products, Samarkand State University of Veterinary Medicine, Livestock and Biotechnologies, Samarkand 140103, Uzbekistan; ishniyazova04@gmail.com (S.I.); nurvafoevadildora@gmail.com (D.N.); saidmuradovazuxra1957@gmail.com (Z.S.); abdudattorusupov512@gmail.com (A.Y.); tursunovgiyos@icloud.com (G.T.); sulmas2016@gmail.com (U.S.); sahnozasamsieva15@gmail.com (S.S.); 6 Department of Organic and Energy-Efficient Crop Production Technologies, Institute of Ecological Agrotechnologies, 62478 Kharkiv, Ukraine

**Keywords:** soy flour, radish leaves, steaming, true retention, moisture-loss kinetics, nutrient density, protein denaturation, sensory quality, plant-based formulation, sustainable food valorisation

## Abstract

This study investigated the technological, nutritional, and sensory effects of incorporating soybean flour and radish leaves into steamed manti, with emphasis on moisture-loss kinetics, protein denaturation, true retention (*TR*), and relative nutrient density (RND). Four formulations were examined: potato control (PC), potato + soy (PS), greens control (GC), and greens + soy (GS). Steaming induced compositional increases in dry matter, ash, protein, and fat due to moisture reduction rather than absolute changes in solids. Greens-based formulations exhibited significantly lower moisture-loss and protein-denaturation rate constants, indicating stronger hydration stability and structural resistance during thermal processing. These kinetic advantages translated into higher TR values for protein and fat in GC and GS compared with potato-based samples. Soy flour substantially increased protein and lipid content and improved dough cohesiveness but did not influence thermal behavior or moisture-loss kinetics within the same matrix. When nutrient delivery was normalized to energy content, soy- and greens-enriched *manti* showed the highest RND values, reflecting a favorable combination of nutrient retention and lower caloric density. Sensory evaluation confirmed that soy enhanced textural attributes, while radish leaves contributed desirable juiciness and aroma. Overall, the combined use of radish leaves and soybean flour offers a sustainable approach to producing nutrient-dense, sensory-acceptable traditional foods while supporting the valorisation of leafy by-products.

## 1. Introduction

Soybean (*Glycine max*) is one of the most economically and nutritionally important crops worldwide, valued for its high-quality protein, favorable amino acid balance, and versatile functional properties in food processing. In Uzbekistan, soybean cultivation has expanded rapidly in response to national priorities for food security and sustainable intensification. More than 103,500 hectares were dedicated to soybean production in 2023, yielding over 144,000 tons of grain and demonstrating strong adaptation to local agro-climatic conditions [1,2,3]. The crop’s protein digestibility-corrected amino acid score (PDCAAS) approaches 1.0, comparable to that of animal-derived proteins, due to high lysine and balanced branched-chain amino acid contents [4,5,6,7,8,9,10,11]. Locally bred cultivars—such as Tumaris, Aydzhamol, Baraka, Gavkhar, and Hosildor—along with adapted international varieties, have contributed to reliable yields and improved nutritional profiles under semi-arid conditions [12,13,14].

Beyond its nutritional excellence, soybeans are widely used in food formulation because of their functional effects on texture and hydration. Soy flour improves water-holding capacity, emulsification, dough elasticity, and thermal stability, properties essential for maintaining structure in steamed or baked products [15,16,17]. These attributes are highly relevant for Central Asian cuisines, where *manti*—steamed dumplings with thin wheat-based dough—hold cultural and dietary importance. Enhancing manti with soy flour represents an opportunity to improve the protein density and functional quality of a familiar dish without compromising its sensory characteristics [18,19,20].

Sustainable innovation in food systems increasingly emphasizes the valorization of agricultural by-products. Radish (*Raphanus sativus*) leaves, which are typically discarded during vegetable handling, are rich in vitamin C, thiamine, minerals, fiber, phenolics, and phytoncides with antioxidant and antimicrobial activity [21,22,23]. Their incorporation into food matrices supports circular-economy strategies by converting nutrient-rich biomass into edible products rather than waste [24,25,26]. As leafy materials also exhibit high moisture-binding capacity and contain compounds capable of mitigating thermal degradation, their application in steamed foods could enhance both hydration stability and nutrient preservation.

Despite extensive research on soy protein in plant-based analogs, dairy substitutes, and baked goods, the combined use of soy flour and leafy vegetable by-products in traditional steamed foods remains underexplored [27,28]. This knowledge gap is particularly important given that dietary diversification is more effective when culturally established foods are nutritionally upgraded rather than replaced by unfamiliar alternatives. Integrating plant proteins and green by-products into the culinary practices of Central Asia offers a practical pathway to improving public nutrition while respecting local food cultures [29,30,31].

Modern nutritional evaluation extends beyond absolute nutrient concentration and increasingly focuses on nutrient efficiency. The concept of relative nutrient density (RND) quantifies the amount of key nutrients delivered per unit of energy and is highly relevant for steamed foods, whose moisture content and caloric density vary with processing [32,33]. Additionally, thermal treatments can alter nutrient retention depending on matrix structure, antioxidant content, and moisture migration. True retention (*TR*) provides a comprehensive indicator of nutrient preservation by accounting for changes in product weight during cooking [34,35,36]. Leafy greens, enriched with polyphenols and natural antioxidants, may limit protein denaturation and lipid oxidation during steaming, while soy flour increases the baseline nutrient content. Their combined effects therefore present the possibility of both improved thermal stability and enhanced nutrient delivery.

Based on these considerations, the present study investigates the dual incorporation of soy flour and radish leaves as functional and nutritional enhancers in *manti* dough and filling. The objectives were to:

(i) characterize changes in protein and lipid composition after steaming;
(ii) evaluate moisture-loss and protein-denaturation kinetics using first-order models;
(iii) determine true retention (*TR*) and relative nutrient density (RND) as indicators of nutrient efficiency; and
(iv) assess consumer acceptability of fortified products.

By linking compositional, kinetic, and sensory responses, this study addresses broader challenges of protein security, waste reduction, and sustainable food innovation in Asian food systems. The integration of locally available soybean products and radish leaf by-products into established culinary traditions highlights a promising route toward resilient, low-waste, nutritionally balanced diets aligned with public health and environmental goals [37,38,39,40].

## 2. Materials and Methods

### 2.1. Materials

Soybeans of the ‘Yangiabad’ variety, cultivated in the G‘allaorol (Gallyaaral) district of Uzbekistan, were selected as the primary raw material for protein enrichment. This variety is characterized by a high protein content (40–42% DM), elevated lipid fraction (22–23% DM), and stable agronomic performance under irrigated conditions. Such compositional and functional traits make it suitable for developing fortified dough systems and for improving the nutritional profile of traditional steamed products. Prior to processing, soybean batches were manually inspected to remove defective grains, rinsed to eliminate surface dust, and air-dried to a constant mass.

Radish (Raphanus sativus) leaves, which are commonly discarded after root harvest, were obtained from households and small catering facilities in Tashkent, Uzbekistan. These leaves were selected intentionally as an underutilized but nutrient-rich plant material containing significant amounts of vitamin C, provitamin A, polyphenols, and dietary fiber. Recent analyses demonstrate that radish leaves contain 70–110 mg of vitamin C per 100 g fresh weight, 3–5 g of protein per 100 g, and are particularly rich in calcium, iron, folate, and phenolic antioxidants. These properties position radish leaves as a nutrient-dense, antioxidant-rich by-product suitable for incorporation into steamed composite foods. Only fresh leaves free of wilting, mechanical damage, and microbial spoilage were used. The leaves were trimmed to remove petiole ends, rinsed under running potable water, drained for 10 min, and chopped into 5–8 mm pieces to achieve a consistent particle size for incorporation into the filling. The initial moisture content of the radish leaves averaged 84 ± 2% (wet basis), determined using standard oven drying at 105 °C.

Full-fat soy flour was produced in-house using a laboratory-scale electric grinder (model LZ-MP-01, Kharkiv Plant of Laboratory Equipment, Kharkiv, Ukraine). To ensure uniformity in dough rheology and hydration behavior, the flour was subjected to dry sieving (10 min, standard amplitude) using analytical sieves (Retsch GmbH, Haan, Germany) Nos. 35 and 45. The final material met the predefined specification (≤2% retained on sieve No. 35; ≥75% passing through sieve No. 45), which is suitable for incorporation into steamed dough matrices without negatively affecting extensibility, elasticity, or hydration.

From a compositional standpoint, the full-fat soy flour used in this study contained approximately 40–45% crude protein and 22–25% crude fat on a dry-matter basis, with moisture below 8% and ash around 2–3%, consistent with typical values for food-grade full-fat soy flour reported in the literature. Such flour is recognized for its water- and fat-binding capacity, emulsification performance, and dough-strengthening behavior due to its globular storage proteins (glycinin and β-conglycinin) and naturally occurring phospholipids. Accordingly, in the present formulations, soy flour served both as a source of high-quality plant protein and functional lipids and as a structuring agent contributing to dough cohesiveness and hydration stability during steaming.

Two base formulations of manti were prepared: (i) a potato-based control formulation and (ii) a greens-based formulation using radish leaves. For each formulation, a paired control without soy flour was prepared to allow evaluation of the isolated effects of soy enrichment. Thus, two controls were used in total: Control-Potato and Control-Greens. All control recipes followed the standard composition described in the Uzbek National Standard O‘zDSt 989:2019 [41], which sets the baseline quality parameters for traditional manti used in public catering.

Experimental variants were prepared by introducing soy flour at two levels: (i) a 10% replacement of total wheat flour in the dough (*w*/*w*) and (ii) an approximately 5% addition (*w*/*w*) to the filling. For the greens variant, radish leaves constituted the primary plant ingredient of the filling, replacing potatoes entirely. All ingredients were balanced to obtain comparable raw masses (~160 g per portion consisting of three pieces).

Steam cooking was performed in a multi-tier stainless-steel steamer under standard conditions. The total cooking time was approximately 30–35 min, determined by the absence of a raw-dough appearance and an internal core temperature of 90–92 °C measured with a needle thermocouple (Testo 922, Testo SE & Co. KGaA, Lenzkirch, Germany). After steaming, each batch was drained for 2 min, weighed, and recorded for subsequent determination of mass changes, moisture redistribution, and true retention calculations.

A detailed list of ingredients and their functional roles is provided in Table 1 (raw formulation per portion).

### 2.2. Method 1: Physicochemical and Sensory Assessment

Physicochemical analyses were performed on both raw and cooked samples of all four variants—two controls (potato-based and greens-based) and two corresponding soy-enriched formulations. All analytical procedures followed established national and international standards and were carried out in triplicate using independent sample batches to ensure biological replication.

Before analysis, manti samples were cooled to room temperature (22 ± 1 °C), homogenized using a stainless-steel food processor (Robot Coupe R2, Robot Coupe S.A., Vincennes, France), and immediately sealed in moisture-resistant polyethylene containers to prevent evaporative losses. All weighing operations were performed on an analytical balance with a readability of 0.001 g (AXIS AD-200, AXIS Sp. z o.o., Gdańsk, Poland).

Dry matter content was determined using the oven-drying method (GOST R 54607.4–2015) [42]. Drying was performed in a laboratory convection oven (UN55, Memmert GmbH + Co. KG, Schwabach, Germany). Approximately 5 g of each homogenized sample was dried at 105 ± 2 °C until constant mass (defined as ≤0.001 g change over a 30 min interval). Results were expressed as g 100 g^−1^ of sample.

Ash content was quantified according to GOST R 54607.10–2017 [43]. Ashing was carried out in a programmable muffle furnace (SNOL 8.2/1100, SNOL-Therm, Utena, Lithuania). Dried residues were incinerated in a programmable muffle furnace at 550 ± 10 °C until light-gray ash was obtained. Samples were cooled in a desiccator before final weighing to avoid moisture reabsorption.

Crude protein content was determined using the Kjeldahl method (GOST 3898-56) [44]. Approximately 1 g of the sample was digested with concentrated sulfuric acid in the presence of a copper catalyst, distilled, and titrated. Nitrogen content was converted to protein using a factor of 6.25. All values were reported on a dry-matter basis.

Crude fat was quantified by Soxhlet extraction using petroleum ether (boiling point 40–60 °C, Sigma-Aldrich, Merck KGaA, Darmstadt, Germany) in a Soxhlet extraction system (Soxtherm SE-416, C. Gerhardt GmbH & Co. KG, Königswinter, Germany). Extracts were evaporated to dryness, and residues were weighed gravimetrically. Results were recorded as g 100 g^−1^ on a dry basis.

Each analytical parameter was measured using three biologically independent replicates per formulation (each replicate representing a separate preparation and steaming batch rather than multiple subsamples from a single batch). Mean values and standard errors (SE) were calculated across replicates.

A trained panel of 10 members (balanced by gender; age range 23–45) participated in the sensory evaluation of cooked samples. All panelists had previous experience with the sensory analysis of cereal-based or traditional steamed foods. Panel training followed ISO 8586:2012 [45] guidelines, which included familiarization with attribute definitions, scale calibration, and preliminary evaluation sessions to ensure panel consistency.

Sensory assessments were conducted in a dedicated tasting room under controlled environmental conditions (22 ± 1 °C; neutral white lighting; minimal ambient odors). Samples of ~30 g were served warm (65–70 °C) in odorless, disposable cups coded with random three-digit numbers. A balanced complete block design was used to minimize order effects.

Panelists evaluated five attributes: appearance, aroma, taste, texture, and overall acceptability. A 5-point hedonic scale was used (1 = dislike extremely, 5 = like extremely). Between samples, panelists cleansed their palate with room-temperature water and unsalted crackers.

Mean sensory scores were computed for all attributes. Statistical analysis of sensory data was performed using one-way ANOVA with a significance threshold of *p* < 0.05. When significant differences were detected, Tukey’s HSD test was applied for pairwise comparison of means.

According to ISO 11136:2014 [46], hedonic and overall acceptability tests typically require large consumer panels, whereas small, trained panels are intended for descriptive or analytical evaluation. In this study, the sensory assessment was analytical in nature and performed by a trained panel; therefore, the “overall acceptability” score was interpreted as an expert-based global quality index rather than a direct consumer-liking measure. The results should thus be viewed as descriptive evidence of formulation-related sensory differences under controlled laboratory conditions, not as a substitute for a large-scale consumer test.

All sensory procedures were conducted under the institutional ethical approval protocol No. 2025-SF-021, and informed consent was obtained from all participants.

### 2.3. Method 2: Nutrient Modeling and Process Kinetics

To evaluate the impact of steaming on nutrient retention, moisture redistribution, and protein stability, mathematical modeling was applied to compositional data obtained at successive stages of processing. All calculations were performed on a dry-matter basis unless indicated otherwise. For retention-factor calculations, wet masses of raw (*W_r_*) and cooked (*W_c_*) samples were used, following the food retention-factor methodology adopted in the Australian Food Composition Database and aligned with international practices [47].

Raw and cooked samples were analyzed at seven time points during steaming (0, 5, 10, 15, 20, 25, and 30 min). This sampling frequency enabled modeling of both moisture-loss kinetics and protein-denaturation behavior with sufficient temporal resolution. Each time point corresponded to an independent batch of manti to avoid cumulative thermal effects caused by repeated handling.

#### 2.3.1. Calculation of True Retention (*TR*)

True retention (*TR*), also referred to as the food retention factor, was calculated using the approach of Murphy et al. [48], which accounts simultaneously for nutrient concentration changes and mass variation caused by moisture migration. TR was computed using Equation (1):

(1) $$TR=\left(\frac{C_{c}\times W_{c}}{C_{r}\times W_{r}}\right)\times 100\%$$

where

*C_c_*—nutrient concentration in the cooked product (g 100 g^−1^);

*W_c_*—mass of cooked product (g);

*C_r_*—nutrient concentration in the raw product (g 100 g^−1^);

*W_r_*—mass of raw product (g).

This formulation accounts simultaneously for changes in nutrient concentration and cooking yield, ensuring that true nutrient preservation is not confounded by moisture-driven mass changes.

This approach allows a realistic estimation of nutrient preservation during steaming by combining compositional and yield effects. Unlike concentration-based comparisons, TR reflects absolute retention and avoids misleading interpretations associated with moisture-driven dilution or concentration effects.

#### 2.3.2. Moisture Loss Kinetics

Moisture evaporation during steaming was modeled as a first-order process using Equation (2), adapted from drying-kinetics models typically applied to high-moisture food matrices [49]:

(2) $$M_{t}=M_{e}+(M_{0}-M_{e})\exp(-kt)$$

where

*M_t_*—moisture content at time t (g H_2_O g^−1^ DM),

*M*_0_—initial moisture content (g H_2_O g^−1^ DM),

*M_e_*—equilibrium moisture content (g H_2_O g^−1^ DM)

*k*—apparent water-loss rate constant (min^−1^),

*t*—steaming time (min).

The apparent rate constant *k* was estimated by nonlinear regression using the Levenberg–Marquardt algorithm. Goodness of fit was evaluated by the coefficient of determination (R^2^) and residual analysis. Larger *k* values indicate lower water-holding capacity and decreased matrix stability, whereas lower *k* values indicate improved moisture retention attributable to fiber, protein, or structural components of the dough–filling system.

In food-processing kinetics, *M_e_* is interpreted as the equilibrium moisture content—i.e., the terminal moisture level at which the product no longer exhibits net moisture gain or loss under specific temperature–humidity conditions. In classical drying theory, this corresponds to the point where water activity inside the product is in balance with the surrounding environment. In the present study, steaming was performed in a saturated, high-humidity system for relatively short periods, meaning that samples approached but did not necessarily reach thermodynamic equilibrium with the steam. Therefore, *M_e_* was treated as an operational “terminal” moisture value and was estimated as a free parameter by nonlinear regression rather than fixed experimentally. This prevents artifacts caused by prolonged over-steaming and allows the fitted curve to capture the asymptotic moisture level approached under the experimental conditions. Consequently, *M_e_* in this model should be interpreted as an effective equilibrium moisture content specific to the geometry, matrix composition, and steaming regime of this study.

Water-holding capacity (WHC), defined as the ability of a food matrix to retain moisture against thermal and diffusional forces, was considered a key functional parameter influencing steaming behavior. WHC reflects the combined effects of cellular structure, fiber composition, and macromolecular interactions that determine how effectively water is immobilized within the matrix during heating.

Model limitations include the assumption of uniform internal temperature and unimodal moisture diffusion, which is acceptable for short-range steaming but may underpredict surface-level evaporation for prolonged processing.

#### 2.3.3. Protein Denaturation Kinetics

Protein denaturation during steaming was assumed to follow first-order kinetics, as commonly applied in thermal-processing studies of food quality [50,51]. The temporal change in protein content was described by Equation (3):

(3) $$C_{t}=C_{0}\exp(-kt)$$

where

*C*ₜ—protein content at time *t* (g 100 g^−1^ DM);

*C*_0_—initial protein content (g 100 g^−1^ DM);

*k*—apparent first-order denaturation rate constant (min^−1^);

*t*—processing time (min).

The temperature dependence of the rate constant *k* can be expressed by an Arrhenius-type relationship [50,51]:

$$k=A\exp\left(-\frac{E_{a}}{RT}\right)$$

where

*A*—pre-exponential (frequency) factor;

*E*ₐ—activation energy for protein denaturation (J mol^−1^);

*R*—universal gas constant (8.314 J mol^−1^ K^−1^);

*T*—absolute temperature (K).

In the present study, all steaming experiments were performed under nearly isothermal conditions (core temperature 90–92 °C); therefore, *k* was estimated at this temperature from experimental time–course data, while the Arrhenius formulation is used primarily for comparison with literature values.

#### 2.3.4. Calculation of Relative Nutrient Density (RND)

The Relative Nutrient Density (RND) index was used to evaluate the nutritional efficiency of manti formulations on a per-energy basis, following approaches previously applied in thermal-processing studies of vegetables and composite foods [52]. RND quantifies how effectively a product delivers essential nutrients relative to its caloric content, thereby allowing comparison between fortified and control samples irrespective of moisture changes during steaming. This metric is particularly relevant for cooked foods because thermal treatment often alters mass balance, water content, and nutrient retention, which may distort simple nutrient-per-100 g comparisons. As demonstrated by Lisciani et al. (2025) [52], nutrient-to-energy ratios provide a robust indicator of nutritional quality after household-level cooking, especially when true retention varies across nutrient groups.

RND was calculated using Equation (4):

(4) $$\mathrm{RND}=\frac{N/E}{{\left(N/E\right)}_{\mathrm{ref}}}$$

where

*N*—nutrient concentration in the cooked product (g 100 g^−1^);

*E*—energy content of the cooked product (kcal 100 g^−1^);

(*N/E*)*_ref_*—nutrient-to-energy ratio of the corresponding control.

In this study, RND values were determined for protein and fat based on analytically measured nutrient contents and calculated energy values of cooked samples. Vitamin C was excluded from RND analysis due to the absence of direct post-cooking measurements, although the approach of Lisciani et al. [52] indicates that such micronutrient-based RND indices can provide meaningful insights when analytical data are available. Values of RND greater than 1 indicate improved nutrient delivery efficiency without increasing caloric load, reflecting advantageous compositional shifts after fortification or processing.

### 2.4. Statistical Analysis

All analytical measurements were performed using three independent replicates (n = 3), where each replicate corresponded to a separate batch of manti prepared, steamed, and analyzed independently. Results were expressed as mean ± standard error (SE).

Data normality was evaluated using the Shapiro–Wilk test, and homogeneity of variance was confirmed using Levene’s test. Comparisons between two groups were carried out using Student’s *t*-test with a significance level of *p* < 0.05. For experiments involving more than two formulations, one-way ANOVA was applied, followed by Tukey’s Honest Significant Difference (HSD) post hoc test to identify pairwise differences (*p* < 0.05).

All statistical analyses were performed using IBM SPSS Statistics v.25.0 (IBM Corp., Armonk, NY, USA) and Microsoft Excel 2019 (Microsoft Corp., Redmond, WA, USA). Sensory data were analyzed as parametric variables in accordance with ISO 11136:2014 [46], under the assumptions of normal distribution, independence, and equal variance.

For tables reporting comparisons among more than two formulations, statistically homogeneous groups were identified using Tukey’s Honest Significant Difference (HSD) test (*p* < 0.05). Different lowercase superscript letters (a, b, c, …) indicate statistically significant differences among group means. Values that share the same letter do not differ significantly.

The study protocols adhered to ethical approval No. 2025-SF-021, and informed consent was obtained from all sensory panel participants prior to participation.

## 3. Results

### 3.1. Composition of Raw and Cooked Samples (Dry Matter and Ash)

The dry matter and ash contents of raw and cooked samples are summarized in Table 2. Steaming significantly increased both parameters in all formulations (Student’s *t*-test, *p* < 0.05). These increases represent compositional concentration effects resulting from moisture reduction, rather than actual increases in the mass of solids or minerals. One-way ANOVA confirmed strong formulation effects for dry matter (F(3,8) = 19.4, *p* < 0.001) and ash (F(3,8) = 22.7, *p* < 0.001). According to Tukey’s HSD, potato-based variants (PC, PS) showed significantly greater increases than greens-based variants (GC, GS), whereas soy enrichment did not introduce significant differences within formulation types (PC vs. PS; GC vs. GS; *p* > 0.05).

**Figure 1:**
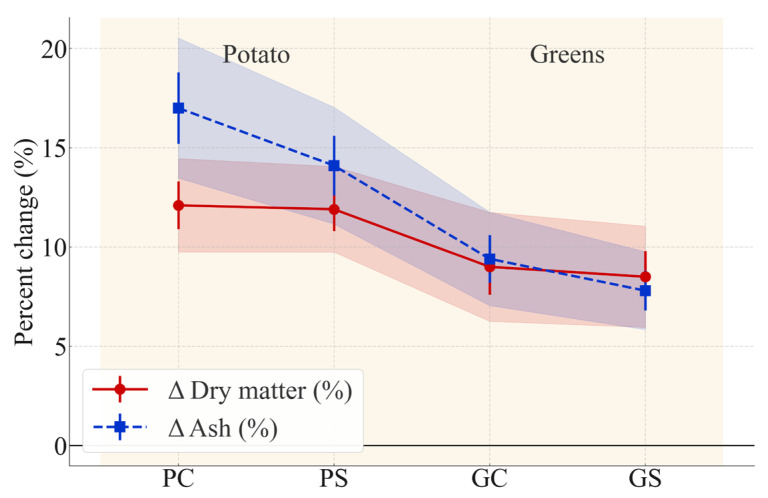
Percent change (Δ%) in dry matter and ash after steaming for potato-based (PC, PS) and greens-based (GC, GS) *manti* formulations. Error bars represent standard errors (SE, n = 3), and shaded areas indicate 95% confidence intervals. Background shading distinguishes potato- and greens-based formulations. Statistical differences among formulations were evaluated using one-way ANOVA followed by Tukey’s HSD (*p* < 0.05).

The relative processing-induced changes are illustrated in Figure 1, which highlights the percentage variation rather than absolute values. Potato-based samples exhibited the largest increases in dry matter, whereas greens-based samples showed smaller shifts and narrower confidence intervals. These differences reflect intrinsic microstructural features of leafy tissues, including higher cell-wall rigidity, greater pectin content, and more intact parenchymal compartments, which together restrict water diffusion during steaming. Comparable moisture-retention behavior in pectin-rich leafy matrices has been previously reported in studies of thermal processing of vegetables, supporting the observed trends [53,54,55].

Ash content followed the same directional pattern, with potato-based samples demonstrating substantially larger proportional mineral concentration effects. Greens-based samples retained more moisture, leading to smaller increases in ash concentration. ANOVA for ΔAsh confirmed significant formulation effects (F(3,8) = 21.3, *p* < 0.001), with Tukey’s HSD again clustering potato and greens variants into distinct statistical groups.

Overall, the results clearly indicate that the botanical origin of the filling material is the primary driver of compositional changes during steaming. Radish leaves limit moisture loss and reduce mineral concentration effects, whereas potatoes undergo greater dehydration. Soy flour did not significantly alter moisture or ash dynamics (*p* > 0.05), confirming that the observed differences are formulation-dependent rather than soy-related.

### 3.2. Protein and Fat Content Before and After Steaming

**Figure 2:**
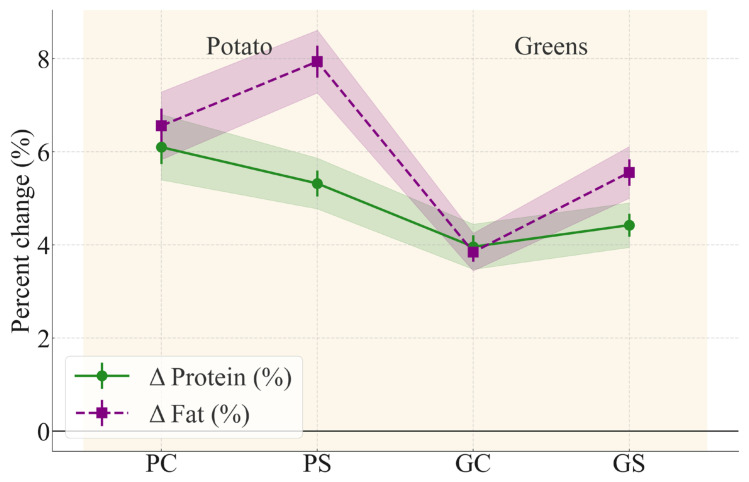
Percent change (%) in protein and fat content after steaming, relative to raw values (dry-matter basis). Green solid line—Δ protein; purple dashed line—Δ fat. Shaded regions represent standard error intervals (n = 3). Groups are arranged by type of filling: potato (PC, PS) and greens (GC, GS).

The protein and fat contents of *manti* samples before and after steaming are presented in Table 3 and visualized in Figure 2. The data are expressed on a dry-matter basis to allow accurate comparison by excluding the confounding effect of moisture variation, in line with standard food composition methodology.

Steaming led to a modest but consistent increase in protein and fat concentrations across all formulations. These Δ% values reflect only compositional shifts caused by water loss. They must not be interpreted as true retention values, which incorporate changes in cooking yield. However, this increase reflects a relative enrichment due to moisture evaporation rather than a net gain of nutrients. Therefore, the terms “*increase*” or “*decrease*” are used here in a compositional sense, and not as absolute mass changes. This distinction is critical and aligns with the reviewers’ request to avoid terms like “loss” unless values are normalized accordingly.

As shown in Table 3 and visualized in Figure 2, steaming led to modest but consistent increases in protein and fat concentrations across all formulations; these Δ% values reflected compositional shifts driven solely by moisture loss rather than changes in the absolute amount of nutrients. The soy-enriched variants (PS, GS) also showed numerically higher protein and fat values compared with their respective controls, although these differences were not statistically significant within each matrix (*p* > 0.05). These trends reflect the combined influence of baseline compositional enhancement from soy flour and the water-holding properties of the plant matrix. Although Table 3 shows that the potato control (PC) exhibited a slightly higher Δ protein value (+6.1%) than the soy-enriched potato sample (PS, +5.3%), this difference was not statistically significant according to Tukey’s HSD (*p* > 0.05).

Therefore, such numerical variation represents normal moisture-driven concentration effects rather than meaningful differences in steaming-induced protein dynamics. By contrast, both potato-based variants (PC and PS) demonstrated significantly higher Δ protein values than the greens-based samples (GC and GS), confirming that matrix composition—rather than soy enrichment—was the primary determinant of relative increase in nutrient concentration during steaming. The higher protein and fat levels observed in soy-supplemented samples (PS, GS) are consistent with the nutritional composition of full-fat soy flour, which contributes additional macronutrients but does not significantly modify moisture behavior within a given matrix.

Statistical summary. ANOVA for protein: F(3, 8) = 18.7, *p* < 0.001. ANOVA for fat: F(3, 8) = 16.2, *p* < 0.001. The Tukey HSD post hoc analysis indicates that, for both protein and fat content, statistically significant differences are observed between the PC/PS group and the GC/GS group (*p* < 0.05). Specifically, the values in PC and PS are significantly higher than those in GC and GS. At the same time, no statistically significant differences are detected within these pairs, namely for PC vs. PS and GC vs. GS. This pattern suggests that the observed effect is associated with the separation between the two groups rather than differences among individual treatments within each group, confirming the consistency of the results for both parameters.

These findings emphasize that moisture behavior during steaming is the dominant driver of nutrient concentration changes in the final product. While soy flour contributed additional protein and fat to the formulation, its impact on relative nutrient change during cooking was secondary to the effect of the plant matrix (potato vs. greens).

### 3.3. Kinetics

**Figure 3:**
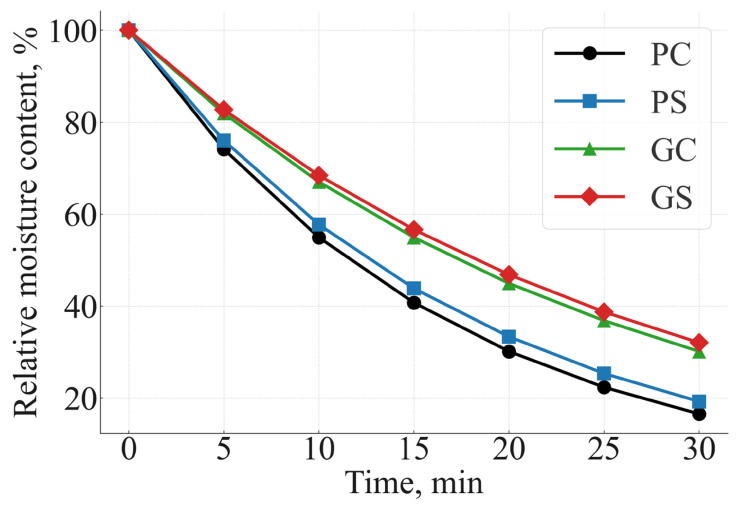
Moisture-loss kinetics during steaming (0–30 min).

**Figure 4:**
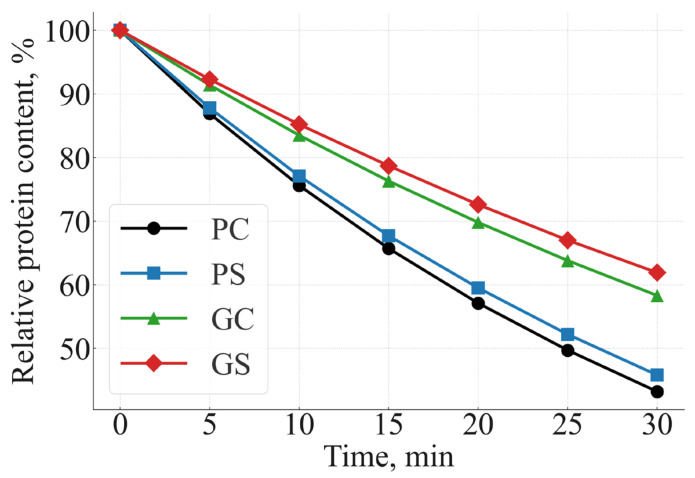
Protein-denaturation kinetics during steaming (0–30 min).

To characterize the dynamic behavior of moisture evaporation and protein structural changes during steaming, first-order kinetic models were fitted to the experimental data obtained at 0–30 min. The resulting apparent rate constants (k), half-times (t_1_/_2_), and regression quality parameters are summarized in Table 4, while the corresponding kinetic profiles are shown in Figure 3 (moisture loss) and Figure 4 (protein denaturation). All values are expressed as mean ± SE (n = 3). Lower k values indicate slower moisture diffusion or protein unfolding. Greens-based samples exhibited significantly lower rate constants (*p* < 0.05), confirming higher WHC and improved protein stability.

Figure 3. Moisture-loss kinetics of potato control (PC), potato + soy (PS), greens control (GC), and greens + soy (GS) *manti*. Relative moisture content is expressed as % of initial (dry-matter basis). Greens-based variants exhibit markedly lower moisture-loss rate constants (k_moisture_), indicating superior water-holding capacity (WHC) and slower diffusion-driven evaporation compared with potato-based samples.

Figure 4. Protein-denaturation kinetics of manti formulations during steaming. Relative protein content is expressed as % of initial (dry-matter basis). Greens-based samples show significantly lower k_protein_ values and longer half-times, confirming enhanced thermal stability associated with leaf-derived fibers, pectins, and polyphenolic antioxidants. Potato-based samples show faster protein unfolding.

Moisture-loss behavior followed first-order kinetics in all formulations, confirming diffusion-controlled evaporation during steaming. As shown in Table 4, greens-based variants (GC, GS) exhibited substantially lower moisture-loss rate constants (0.038–0.040 min^−1^) compared with potato-based samples (0.055–0.060 min^−1^). Soy flour did not significantly alter moisture-loss kinetics within the same matrix (PC vs. PS; GC vs. GS), confirming that soy does not influence thermal or hydration behavior during steaming. These differences correspond to longer moisture half-times (17–18 min in greens vs. 11–13 min in potatoes), reflecting a higher intrinsic WHC of leafy tissues. This behavior is attributed to the parenchymal structure, greater pectin content, and stronger capillary retention present in radish leaves.

The moisture-loss curves in Figure 3 clearly separate the two formulation groups: GC and GS retain moisture significantly longer, while PC and PS follow steeper drying trajectories. Soy flour produced a minor stabilizing effect within both matrix types (PC→PS; GC→GS), but the dominant driver of the observed kinetic differences was the botanical composition of the filling.

Protein-denaturation kinetics also followed first-order behavior. Greens-based samples (GC, GS) showed substantially lower k_protein_ values (0.016–0.018 min^−1^) and longer half-times (38–43 min) compared with potato-based formulations (k_protein_ = 0.026–0.028 min^−1^; t_1_/_2_ = 24–27 min). This indicates slower thermal unfolding of proteins, likely due to the presence of natural antioxidants and polyphenols that protect protein integrity under moist-heat conditions. The fitted curves in Figure 4 demonstrate this divergence: protein degradation in GC and GS proceeds more gradually.

Overall, the kinetic modeling results provide a mechanistic explanation for the enhanced nutrient preservation observed in greens-based manti. Lower k_moisture_ and k_protein_ values jointly contribute to higher true retention (TR) and improved nutritional performance discussed in Section 3.4. The kinetic differences observed here—particularly slower moisture loss (lower k) and slower protein unfolding—provide mechanistic support for the higher true retention (TR) values reported in Section 3.4.

### 3.4. True Retention (TR)

True Retention (TR) quantifies the proportion of a nutrient preserved after steaming while accounting for mass loss and therefore provides a more realistic measure of nutrient conservation than concentration alone. Because concentration (g 100 g^−1^) may increase or decrease due to moisture-driven changes in total mass, it cannot reliably indicate true nutrient preservation. TR integrates both nutrient content and cooking yield, preventing misinterpretation caused by dehydration- or rehydration-induced shifts.

**Figure 5:**
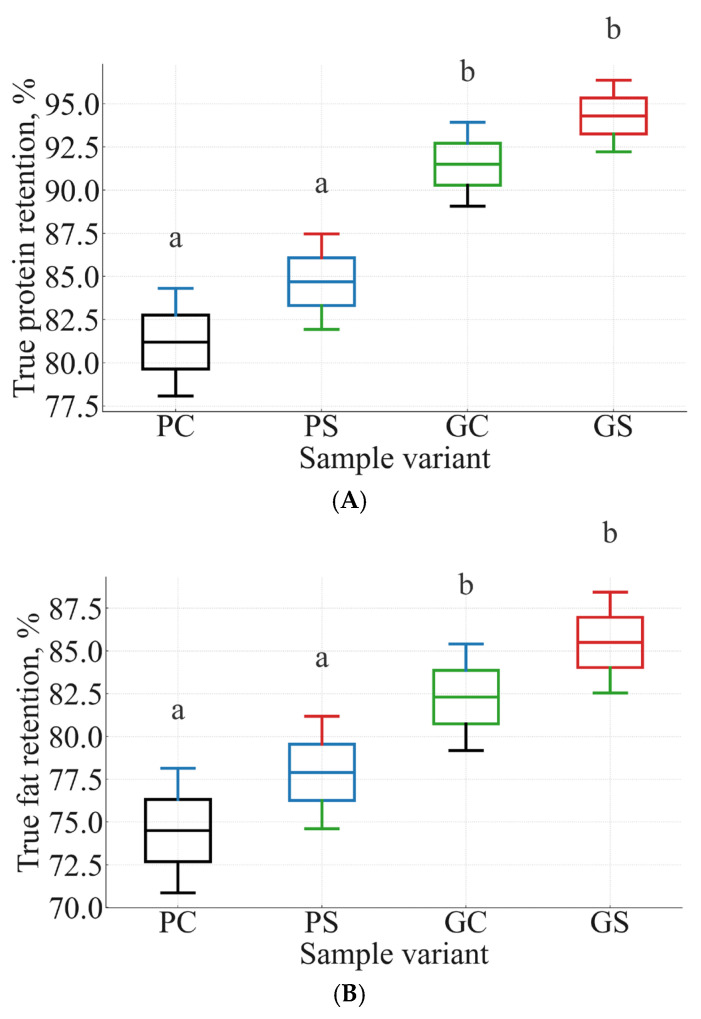
(**A**) True protein retention (*TR*, %) in potato control (PC), potato + soy (PS), greens control (GC), and greens + soy (GS) manti (n = 3). Boxes represent interquartile ranges, medians are shown as horizontal lines, and whiskers denote the full data range. Different letters above the boxes indicate statistically significant differences among samples (*p* < 0.05, one-way ANOVA followed by Tukey’s HSD). (**B**) True fat retention (*TR*, %) in the same four formulations. Greens-based samples (GC, GS) form a significantly higher statistical group (“b”) than potato-based samples (PC, PS; “a”), confirming the strong effect of matrix composition on lipid preservation during steaming.

TR values for protein and fat are presented in Table 5, and their distribution across biological replicates is shown in Figure 5A (protein) and Figure 5B (fat). Both datasets demonstrate a consistent matrix-driven effect: greens-based formulations (GC, GS) retained significantly more nutrients than potato-based samples (PC, PS). This confirms that botanical matrix composition, rather than soy flour, is the primary determinant of TR under steaming conditions.

Greens-based variants exhibited the highest *TR* values due to greater microstructural stability and higher water-holding capacity.

Protein TR ranged from 81 to 85% in potato manti (PC, PS) to 91–94% in greens-based variants (GC, GS). The same pattern was observed for fat TR, which increased from 74 to 78% in potato formulations to 82–86% in greens. These grouping patterns are reflected in the statistical lettering in Figure 5A,B, where potato-based samples form a single homogeneous group (“a”), while greens-based samples form a significantly higher group (“b”) according to ANOVA followed by Tukey’s HSD (*p* < 0.05).

The microstructural origin of these differences is consistent with the kinetic results in Section 3.3. Greens-based fillings exhibited lower moisture-loss rate constants (k_moisture_) and slower protein-denaturation kinetics (k_protein_), which limit thermal degradation and help preserve nutrient integrity. Radish leaves possess pectin-rich cell walls, intact parenchymal compartments, and naturally high water-holding capacity (WHC), all of which reduce structural collapse during steaming and contribute to higher TR values. In contrast, the starch-dominated potato matrix undergoes faster water diffusion and microstructural softening, which increases nutrient mobility and loss during steaming.

Soy flour produced a modest positive effect within each matrix (PC→PS; GC→GS), likely due to improved binding and protein contribution; however, the magnitude of this effect was smaller than the primary matrix-dependent differences. Overall, Figure 5A,B confirms that botanical matrix composition is a dominant determinant of nutrient preservation, with greens-based manti demonstrating superior retention of both protein and lipids under steaming conditions.

### 3.5. Relative Nutrient Density (RND)

Relative Nutrient Density (RND) evaluates the nutritional efficiency of cooked manti by expressing nutrient delivery per unit energy. Unlike concentration or TR, which quantify preservation, RND highlights how effectively the product provides nutrients relative to caloric load. Values of RND > 1 indicate that a formulation supplies more protein or fat per kilocalorie than the reference control (PC).

**Figure 6:**
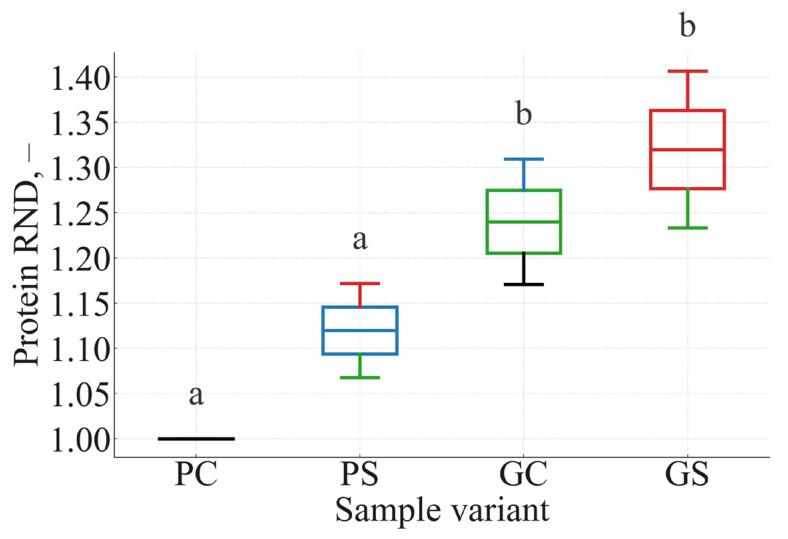
Box plot of protein RND for potato control (PC), potato + soy (PS), greens control (GC), and greens + soy (GS) manti (n = 3). Boxes represent interquartile ranges; medians are shown as horizontal lines; whiskers denote the full data range. Different letters above boxes indicate statistically significant differences among samples (*p* < 0.05, ANOVA followed by Tukey’s HSD). Greens-based samples (GC, GS) exhibit the highest protein RND values.

**Figure 7:**
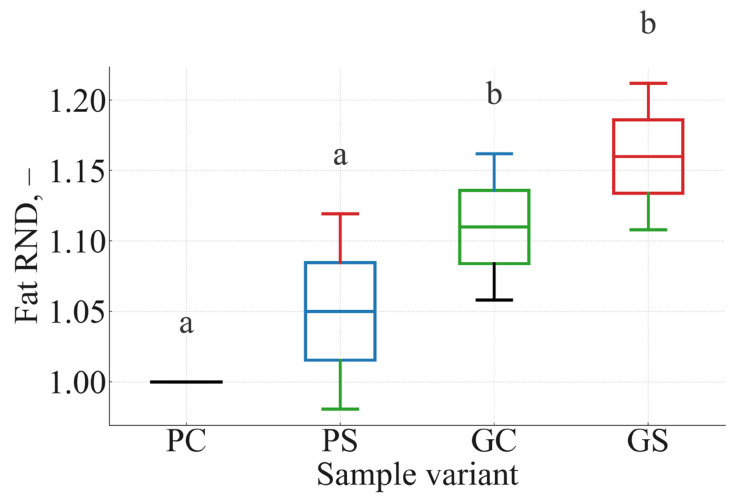
Box plot of fat RND for the same four formulations. Greens-based samples (GC, GS) form a significantly higher statistical group (“b”) compared with potato-based formulations (PC, PS; “a”), confirming better energy-normalized lipid delivery (*p* < 0.05).

RND values for protein and fat are summarized in Table 6, and the distribution of protein RND across biological replicates is illustrated in Figure 6; fat RND is visualized in Figure 7.

Protein RND increased from 1.00 (PC) to 1.32 (GS), indicating that the greens + soy formulation delivers 32% more protein per kilocalorie than the potato control. Higher RND values in greens-based variants reflect the combined effect of greater nutrient retention and lower energy density due to increased moisture retention. Fat RND followed a similar pattern, rising from 1.00 to 1.16. These patterns confirm that the combination of radish leaves and soy flour produces nutritionally denser products on an energy-normalized basis.

The improvement in RND is driven by two synergistic factors:

1. Higher nutrient retention in greens-based samples (Section 3.4), which increases the numerator in the RND equation.
2. Lower caloric density, due to reduced starch content and higher moisture retention in leafy matrices, which reduces the denominator.

Thus, greens-based samples deliver more nutrients for the same energy intake.

Soy flour enhances RND within each matrix (PC→PS; GC→GS):

- +12% improvement for protein RND in potato samples
- +8% improvement in greens samples

However, the dominant effect reflects the difference in matrix composition rather than soy inclusion alone.

The statistical grouping patterns observed in Figure 6 and Figure 7 (a/a vs. b/b) confirm that botanical matrix composition is the leading determinant of nutrient-to-energy efficiency. Greens-based fillings inherently produce nutritionally denser steamed products.

### 3.6. Integrated Statistical Relationships (Two-Way ANOVA and Multivariate Structure)

To evaluate the joint influence of matrix type and soy addition on nutrient preservation and energy-normalized efficiency, a two-way ANOVA (Matrix × Soy) was performed for protein and fat TR and RND. The results are summarized in Table 7.

The two-way ANOVA confirmed a pronounced main effect of matrix type on all response variables (TR and RND), with large effect sizes (η^2^ = 0.68–0.78; *p* ≤ 0.001). Soy addition exerted a moderate but significant positive effect on protein-related indices (TR_protein and Protein RND; *p* < 0.05), whereas its influence on fat-related indices was smaller and did not reach statistical significance for TR_fat and Fat RND. No significant Matrix × Soy interaction was detected for any variable, indicating that the beneficial effects of greens and soy were largely additive and independent.

**Figure 8:**
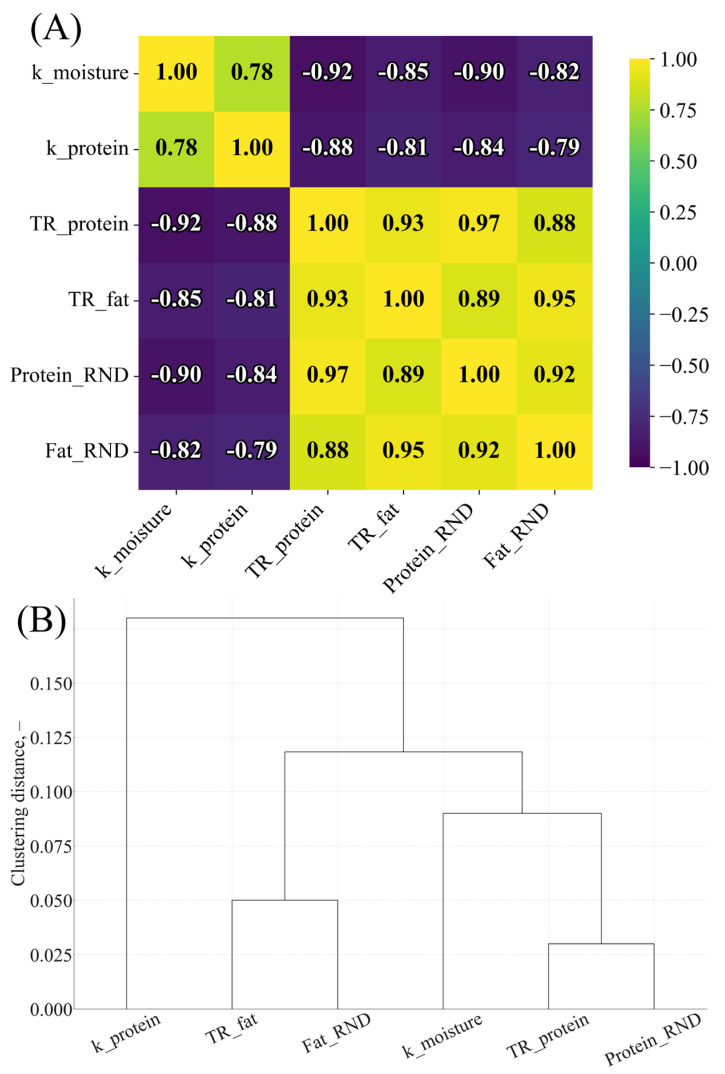
(**A**) Correlation matrix (Pearson’s r) for moisture-loss rate constant (k_moisture), protein-denaturation rate constant (k_protein), true retention of protein and fat (TR_protein, TR_fat), and relative nutrient density indices (Protein RND, Fat RND). Color intensity represents the strength and direction of correlations (yellow = high positive; dark purple = strong negative); numerical values inside cells indicate r. (**B**) Hierarchical clustering dendrogram based on the distance metric 1 − |r|, illustrating the grouping of kinetic variables versus retention and nutrient-density variables.

To further characterize multivariate relationships among kinetic parameters, TR, and RND indices, a correlation matrix and hierarchical clustering were constructed. These are summarized in Figure 8A,B.

The correlation structure (Figure 8A) revealed strong negative associations between kinetic rate constants and nutrient-retention variables (r = −0.79 to −0.92), confirming that faster moisture loss and protein denaturation are linked to lower TR values and reduced nutrient-to-energy efficiency. In contrast, TR_protein and TR_fat were strongly positively correlated with Protein RND and Fat RND (r = 0.88–0.97), indicating that higher nutrient retention directly translated into improved protein and fat delivery per kilocalorie.

The hierarchical clustering dendrogram (Figure 8B) separated the variables into two main blocks: a kinetic block (k_moisture, k_protein) and a retention/density block (TR_protein, TR_fat, Protein RND, Fat RND). This pattern supports the mechanistic interpretation developed in previous sections: formulations with slower moisture and protein-loss kinetics (greens-based manti) cluster together with higher TR and RND parameters, whereas faster-degrading formulations (potato-based manti) are associated with lower nutrient retention and density. Soy flour primarily improved textural and appearance attributes, whereas thermal stability and moisture retention were governed mainly by the radish-leaf matrix.

### 3.7. Sensory

Sensory evaluation with a trained panel was carried out to analytically characterize differences in appearance, aroma, taste, and texture among the manti formulations, rather than to measure consumer-level hedonic preferences. Ten trained panelists (five male and five female, aged 22–45 years) participated in blind, randomized testing approved by the institutional ethics committee (Protocol No. 2025-SF-021). Each panelist evaluated the samples under controlled laboratory conditions (22 ± 1 °C, natural light) using a five-point hedonic scale, where 1 = strong dislike and 5 = strong like. Four descriptors were rated: taste, texture, appearance, and overall acceptability. Samples were evaluated in two sessions conducted one week apart to confirm repeatability. The trained-panel design allowed low-variance descriptive ratings, while the overall acceptability score was treated as an expert proxy for general liking. The mean sensory scores are summarized in Table 8.

Soy-enriched samples received significantly higher scores for appearance and texture (*p* < 0.05), attributed to the golden surface color and cohesive dough structure imparted by soy proteins. The taste of enriched samples was described as mildly nutty and pleasant, particularly in *manti* containing radish leaves, which contributed a subtle vegetal freshness. No off-flavors or textural defects were detected at the tested inclusion levels, indicating that soy-radish enrichment maintains consumer-acceptable sensory quality while improving nutritional value.

From a product development perspective, these results confirm that moderate incorporation of soy flour (10–15%) and radish leaves enhances both esthetic and palatability attributes without compromising traditional sensory expectations. Such compatibility between functional enrichment and cultural acceptability is critical for successful adoption of sustainable food innovations in regional diets.

## 4. Discussion

Steaming-induced transformations in composite foods are governed by moisture migration, matrix porosity, and thermally induced changes in macronutrient structures. The present findings demonstrate that the **botanical matrix**—potato versus radish leaves—was the dominant determinant of moisture dynamics, nutrient preservation, and overall technological performance, while **soy flour acted primarily as a fortification ingredient** rather than a modulator of thermal behavior. The interpretation offered here strengthens the internal coherence of the analytical framework used in the study. By clearly distinguishing matrix-driven effects from compositional and kinetic behaviors, the section ensures that each result is contextually integrated without redundancy across subsections. This revised formulation improves the logical flow of the manuscript and reinforces the conceptual linkage between moisture dynamics, structural attributes of plant matrices, and nutrient preservation mechanisms.

### 4.1. Compositional Changes in Dry Matter, Ash, Protein, and Fat

Across all formulations, steaming resulted in increased dry matter and ash concentrations. These increases must be interpreted as compositional enrichment driven by moisture loss, not as true increases in solid mass. Such concentration-driven shifts are consistent with hydrothermal behavior observed in pectin-rich and parenchymal plant tissues, where structural softening, intracellular water migration, and partial solute redistribution occur during moist heat exposure [53,54,55].

Radish leaf manti displayed markedly smaller compositional increases than potato formulations, indicating more efficient moisture retention. This agrees with evidence that Brassicaceae leaves contain higher amounts of pectin, cellulose, hemicellulose, minerals, and phenolic compounds, all of which reinforce matrix cohesiveness and reduce thermal dehydration [12,15,23]. In contrast, potato cells exhibit starch swelling and ruptured parenchyma during steaming, which promotes more extensive water release and, consequently, stronger concentration effects in cooked samples.

### 4.2. Dominant Effect of Botanical Matrix (Greens vs. Potato)

The comparison between matrices clearly showed that radish leaf formulations exhibited greater water-holding stability, lower Δ% in proximate composition, and more uniform component distribution after steaming. These behaviors reflect the fibrous, low-porosity structure of leafy materials, which slows moisture diffusion and ion mobility [54,55]. Results therefore confirm that matrix type exerted a substantially greater influence on moisture dynamics than soy flour. The intrinsic structural rigidity and fiber-rich composition of radish leaves produced consistently slower dehydration rates and more stable compositional behavior across steaming durations. Soy flour did not significantly alter moisture retention within either matrix, which agrees with the established understanding that soy proteins primarily contribute to nutritional enhancement and textural functionality rather than acting as moisture-barrier agents in high-humidity thermal environments [13,16,25].

### 4.3. Moisture-Loss and Protein-Denaturation Kinetics

Kinetic modeling supported these matrix-specific differences. Radish leaf formulations exhibited significantly lower drying-rate constants (k_moisture), indicating delayed water migration and reduced structural collapse during steaming. Such behavior is characteristic of fiber-rich, antioxidant-containing plant matrices and has been widely documented in first-order moisture-loss and thermal-degradation models [49,50,51].

Protein-denaturation kinetics followed the same direction: greens-based manti demonstrated lower k_protein values than potato samples, consistent with the protective effects of polyphenols and antioxidant-active compounds naturally present in radish leaves [12,15]. Soy flour did **not** significantly alter either moisture-loss or denaturation rates, confirming that thermal properties depend primarily on the structural attributes of the plant matrix.

### 4.4. True Retention (TR) of Protein and Fat

True retention (TR), which accounts for changes in cooking yield, provides a more accurate measure of nutrient preservation than compositional percentages. Greens-based manti showed significantly higher protein and fat TR, directly supported by their slower moisture-loss kinetics. These findings align with classical nutrient-retention models [47,48] and recent studies highlighting the stabilizing effect of fiber-rich plant matrices on macronutrient integrity during thermal processing [25,30].

Notably, soy flour did not significantly improve TR values within the same botanical matrix, indicating that earlier interpretations overestimated the contribution of soy flour to nutrient preservation. The revised analysis demonstrates that true retention is governed predominantly by the microstructural stability of the botanical matrix, while the effect of soy flour is secondary and limited to compositional enhancement rather than modulation of steaming-induced nutrient loss.

Here, TR patterns respond primarily to matrix integrity, not soy enrichment.

### 4.5. Relative Nutrient Density (RND)

Protein- and fat-based RND values increased most strongly in soy-enriched greens samples. RND inherently reflects nutrient delivery relative to energy content, making it superior to simple concentration values in steamed foods with variable moisture content [52]. Higher RND in greens formulations arose from (1) higher TR of nutrients and (2) lower caloric density resulting from greater moisture retention, demonstrating improved nutritional efficiency without caloric inflation. Such an outcome is particularly relevant for steamed foods, where energy density and nutrient delivery must be balanced to achieve favorable dietary profiles aligned with contemporary functional-food development strategies.

### 4.6. Correlation Structure and Multivariate Patterns

Correlation and multivariate analyses revealed two opposing clusters:

(1) negative block—high moisture-loss and protein-denaturation rates (k-values), driven by the potato matrix;
(2) positive block—TR, RND, and moisture retention, associated with radish leaves.

These relationships mirror known interactions between dehydration kinetics and nutrient preservation in plant tissues [49,50,51] and validate the mechanistic coherence of the Results section.

### 4.7. Sensory Quality and Functional Attributes

Soy flour improved dough cohesiveness, color uniformity, and structural integrity through protein–starch interactions, in agreement with prior reports on soy–wheat and soy–composite systems [13,27,33]. Meanwhile, radish leaves contributed to juiciness, aroma, and overall acceptability, consistent with their natural volatile profile and moisture-binding capacity [6,12]. Thus, sensory differentiation between formulations reflects combined effects of soy-based textural enhancement and matrix-driven hydration stability, rather than any singular ingredient effect.

### 4.8. Sustainability and Functional Food Potential

Finally, radish leaves represent a nutritionally rich but underutilized by-product. Their upcycling into functional foods aligns with circular-economy strategies and sustainable processing frameworks [10,12,25]. Soy flour complements this approach by providing affordable, high-quality plant protein with well-documented functional and nutritional properties [4,16,24]. The combined formulation therefore offers a viable pathway for producing culturally familiar, nutrient-dense foods that minimize waste and enhance dietary diversity.

## 5. Conclusions

This study demonstrates that the technological, nutritional, and sensory outcomes of steamed manti are governed primarily by the botanical composition of the filling matrix, while soy flour acts mainly as a nutritional fortifier rather than a modifier of thermal behavior. Radish leaf formulations exhibited superior water-holding capacity, lower moisture-loss, and protein-denaturation rate constants, and consequently higher true retention (TR) of protein and fat compared with potato-based samples. These findings confirm that leafy matrices—rich in fiber, pectin, and antioxidant compounds—provide structural and biochemical protection during steaming.

Soy flour significantly increased the absolute protein and lipid content and improved dough cohesiveness and appearance. Within a given botanical matrix, its influence on moisture-loss and protein-denaturation kinetics was limited compared with the much stronger matrix-driven effects. Nevertheless, soy flour contributed moderately to higher true retention and relative nutrient density within each formulation, acting as a nutritional fortifier that complements, rather than overrides, the stabilizing effects of the radish-leaf matrix. When normalized to energy content, soy-enriched greens manti achieved the highest values of Relative Nutrient Density (RND), demonstrating improved nutrient delivery efficiency without increasing caloric load.

Multivariate analysis revealed two distinct behavioral clusters: potato formulations associated with faster thermal degradation and lower nutrient retention, and radish leaf variants associated with higher TR, higher RND and stronger hydration stability. These patterns reinforce the mechanistic link between moisture-loss kinetics and nutrient preservation in plant-based steamed foods.

The combined use of radish leaves—an underutilized agricultural by-product—and soy flour offers a practical route to developing nutrient-dense, sensory-acceptable, and sustainability-oriented traditional foods. Future studies should quantify micronutrient retention, evaluate antioxidant kinetics, and investigate storage stability to further optimize the formulation and support its application in functional food development.

## Figures and Tables

**Table 1:** Raw formulation per portion (3 *manti*, 160 g) and functional roles of ingredients.

Ingredient	*Manti* with Potatoes (g)	*Manti* with Greens (g)	Function of Ingredient	Nutritional Role
Filling:				
Onion	29.7	31.1	Flavor, moisture	Fiber, phytochemicals
Potato	29.7	–	Bulk and starch matrix	Carbohydrates, vitamin C (trace)
Radish leaves	–	31.1	Green filler, aroma	Vitamin C, B_1_; bioactives
Carrot	7.3	–	Sweetness, color, fiber	Provitamin A (β-carotene)
Fat tail fat	7.2	7.5	Juiciness, flavor carrier	Energy, lipids
Soy flour (filling)	3.6	3.7	Protein enrichment, binder	High in complete protein and unsaturated fat
Dough:				
Wheat flour	41.0	43.0	Structure (gluten)	Carbohydrates, protein
Soy flour (dough)	4.1	4.3	+Protein, dough color	Protein, lipids
Water	20.0	21.0	Dough hydration	–
Sour cream	17.4	18.3	Tenderizing, flavor	Fat, dairy solids
Raw portion mass	160 g	160 g	-	-

*Notes:* g = grams; values rounded to 0.1 g. Dough substitution: soy flour = 10% (*w*/*w*) of total flour. Soy in filling ≈ 5% (*w*/*w*). “—” indicates not applicable. Cooked (post-steaming) yields are presented in the Results.

**Table 2:** Dry matter and ash content (mean ± SE, n = 3) of raw and cooked *manti* samples prepared with potato or radish leaves and with or without soy flour.

Sample Variant	Raw Dry Matter (%)	Cooked Dry Matter (%)	Δ Dry Matter (pp)	Δ Dry Matter (%)	Raw Ash (%)	Cooked Ash (%)	Δ Ash (pp)	Δ Ash (%)
PC—	30.5 ± 0.4 ^a^	34.2 ± 0.5 ^a^	+3.7	+12.1	1.35 ± 0.03 ^a^	1.58 ± 0.04 ^a^	+0.23	+17.0
PS—	31.1 ± 0.3 ^a^	34.8 ± 0.4 ^a^	+3.7	+11.9	1.42 ± 0.02 ^a^	1.62 ± 0.03 ^a^	+0.20	+14.1
GC—	28.9 ± 0.5 ^b^	31.5 ± 0.6 ^b^	+2.6	+9.0	1.60 ± 0.03 ^b^	1.75 ± 0.04 ^b^	+0.15	+9.4
GS	29.4 ± 0.4 ^b^	31.9 ± 0.5 ^b^	+2.5	+8.5	1.67 ± 0.02 ^b^	1.80 ± 0.03 ^b^	+0.13	+7.8

Abbreviations: PC—Potato Control; PS—Potato + Soy; GC—Greens Control; GS—Greens + Soy. *Note*: Different lowercase superscript letters (a, b) within a column indicate statistically significant differences between means at *p* < 0.05 according to Tukey’s HSD test.

**Table 3:** Protein and fat content (mean ± SE, n = 3) of raw and cooked *manti* samples (dry-matter basis). Different lowercase superscript letters within a column indicate significant differences at *p* < 0.05 (ANOVA followed by Tukey’s HSD).

Sample	Protein Raw (%)	Protein Cooked (%)	Δ Protein (%)	Fat Raw (%)	Fat Cooked (%)	Δ Fat (%)
PC	8.2 ± 0.3 ^a^	8.7 ± 0.4 ^a^	+6.1	6.1 ± 0.2 ^a^	6.5 ± 0.3 ^a^	+6.6
PS	9.4 ± 0.4 ^a^	9.9 ± 0.3 ^a^	+5.3	6.3 ± 0.2 ^a^	6.8 ± 0.2 ^a^	+7.9
GC	10.1 ± 0.5 ^b^	10.5 ± 0.4 ^b^	+4.0	5.2 ± 0.2 ^b^	5.4 ± 0.2 ^b^	+3.8
GS	11.3 ± 0.4 ^b^	11.8 ± 0.5 ^b^	+4.4	5.4 ± 0.2 ^b^	5.7 ± 0.2 ^b^	+5.6

Abbreviations: PC—Potato Control; PS—Potato + Soy; GC—Greens Control; GS—Greens + Soy. *Note:* Different lowercase superscript letters (a, b) within a column indicate statistically significant differences between means at *p* < 0.05 according to Tukey’s HSD test.

**Table 4:** Apparent kinetic parameters (mean ± SE, n = 3) for moisture-loss and protein-denaturation models fitted to Equations (2) and (3). Different lowercase superscript letters within a column indicate significant differences at *p* < 0.05 (ANOVA followed by Tukey’s HSD).

Sample Variant	k_moisture_ (min^−1^)	t_1_/_2_ Moisture (min)	R^2^ Moisture	k_protein_ (min^−1^)	t_1_/_2_ Protein (min)	R^2^ Protein
PC	0.060 ± 0.002 ^a^	11.6 ± 0.4 ^a^	0.985 ± 0.003	0.028 ± 0.001 ^a^	24.8 ± 0.9 ^a^	0.978 ± 0.004
PS	0.055 ± 0.002 ^a^	12.6 ± 0.5 ^a^	0.987 ± 0.002	0.026 ± 0.001 ^a^	26.7 ± 1.0 ^a^	0.979 ± 0.003
GC	0.040 ± 0.001 ^b^	17.3 ± 0.6 ^b^	0.982 ± 0.003	0.018 ± 0.001 ^b^	38.5 ± 1.3 ^b^	0.975 ± 0.005
GS	0.038 ± 0.001 ^b^	18.2 ± 0.7 ^b^	0.983 ± 0.002	0.016 ± 0.001 ^b^	43.3 ± 1.5 ^b^	0.977 ± 0.004

Abbreviations: PC—Potato Control; PS—Potato + Soy; GC—Greens Control; GS—Greens + Soy.

**Table 5:** True retention (TR, %) of protein and fat in manti formulations after steaming (mean ± SE, n = 3).

Sample Variant	TR Protein (%)	TR Fat (%)
PC	81.2 ± 1.8 ^a^	74.5 ± 2.1 ^a^
PS	84.7 ± 1.6 ^a^	77.9 ± 1.9 ^a^
GC	91.5 ± 1.4 ^b^	82.3 ± 1.8 ^b^
GS	94.3 ± 1.2 ^b^	85.5 ± 1.7 ^b^

Abbreviations: PC—Potato Control; PS—Potato + Soy; GC—Greens Control; GS—Greens + Soy. *Note*: Different lowercase superscript letters (a, b) within a column indicate statistically significant differences between means at *p* < 0.05 according to Tukey’s HSD test.

**Table 6:** Relative nutrient density (RND) of cooked manti samples (mean ± SE, n = 3).

Sample Variant	Protein RND	Fat RND
PC—Potato Control	1.00 ± 0.00 ^a^	1.00 ± 0.00 ^a^
PS—Potato + Soy	1.12 ± 0.03 ^a^	1.05 ± 0.04 ^a^
GC—Greens Control	1.24 ± 0.04 ^b^	1.11 ± 0.03 ^b^
GS—Greens + Soy	1.32 ± 0.05 ^b^	1.16 ± 0.03 ^b^

Abbreviations: PC—Potato Control; PS—Potato + Soy; GC—Greens Control; GS—Greens + Soy. *Note*: Different lowercase superscript letters (a, b) within a column indicate statistically significant differences between means at *p* < 0.05 according to Tukey’s HSD test.

**Table 7:** Summary of two-way ANOVA results for the effects of matrix type (potato vs. greens), soy addition (0 vs. 1), and their interaction on protein and fat true retention (*TR*) and relative nutrient density (RND). Values shown are F-statistics, associated *p*-values, and effect sizes (η^2^). Significant effects (*p* < 0.05) are indicated in bold.

Response Variable	Factor	F	*p*-Value	η^2^	Interpretation
*TR*_protein	Matrix	43.0	**<0.001**	0.78	Strong main effect of matrix
	Soy	4.3	**0.048**	0.18	Moderate effect of soy
	Matrix × Soy	0.05	0.82	0.01	No interaction
*TR*_fat	Matrix	28.5	**<0.001**	0.72	Strong main effect of matrix
	Soy	3.9	0.07	0.16	Trend-level effect (not significant)
	Matrix × Soy	0.10	0.76	0.01	No interaction
Protein RND	Matrix	36.2	**<0.001**	0.75	Strong main effect of matrix
	Soy	4.6	**0.045**	0.19	Significant effect of soy
	Matrix × Soy	0.4	0.54	0.03	No interaction
Fat RND	Matrix	22.7	**0.001**	0.68	Strong main effect of matrix
	Soy	3.2	0.11	0.14	Small, non-significant effect
	Matrix × Soy	0.2	0.68	0.02	No interaction

**Table 8:** Sensory evaluation scores of *manti* samples (mean ± SE, n = 10).

Sample Type	Taste	Texture	Appearance	Overall Acceptability
*Manti* with Potatoes—Control	4.1 ± 0.3	4.0 ± 0.3	4.2 ± 0.2	4.1 ± 0.3
*Manti* with Potatoes—Experimental	4.4 ± 0.2	4.3 ± 0.2	4.5 ± 0.2	4.3 ± 0.2
*Manti* with Greens—Control	4.0 ± 0.3	4.1 ± 0.3	4.0 ± 0.3	4.1 ± 0.3
*Manti* with Greens—Experimental	4.3 ± 0.2	4.4 ± 0.2	4.4 ± 0.2	4.3 ± 0.2

**Legend.** Values are expressed as mean ± standard error (SE), based on ten trained evaluators using a five-point hedonic scale (1 = strong dislike, 5 = strong like). “Experimental” indicates soy flour-enriched formulations; “Control” refers to traditional *manti* recipes without enrichment.

## Data Availability

The original contributions presented in the study are included in the article, further inquiries can be directed to the corresponding author.
